# Assessing the timing of invasive intervention in NSTE-ACS: insights from a meta-analysis and sequential trial evaluation

**DOI:** 10.3389/fcvm.2025.1712137

**Published:** 2025-11-20

**Authors:** Wei Yang, Xiao-Zhen Ge, Chong-Hui Wang

**Affiliations:** 1 Department of Cardiology, Capital Medical University School of Rehabilitation Medicine, Beijing Bo’Ai Hospital, China Rehabilitation Research Center, Beijing, China; 2 Department of Cardiology, Peking Union Medical College Hospital, Chinese Academy of Medical Sciences & Peking Union Medical College, Beijing, China

**Keywords:** NSTE-ACS, meta-analysis, myocardial infarction, trial sequential analysis, recurrent ischemia

## Abstract

**Background:**

Invasive approaches are commonly recommended for treating patients with non-ST-elevation acute coronary syndromes (*N*STE-ACS) to lower the risk of death caused by myocardial infarction (MI). However, the timing for implementing relevant interventions remains challenging to be determined, largely due to poorly understanding of the long-term clinical outcomes.

**Methods:**

A meta analysis with trial sequential analysis (TSA) was conducted to evaluate the impact of timing on the outcomes of invasive interventions for NSTE-ACS patients. A comprehensive search of PubMed and EMBASE databases identified 14 randomized controlled trials (RCTs), encompassing 16 studies with a total of 9,436 patients, in which two trials have additional long-term follow-up studies. Based on the timing of catheterization, all studies were categorized into two groups: early intervention group (median intervention time <24 h; range from 0.5–9.3 h) and delayed intervention group (median intervention time ≥24 h; range from 18.3–86 h). Clinical outcomes were assessed for primary endpoints (all-cause death or MI) and secondary endpoints (recurrent ischemia, requiring cardiac revascularization or major bleeding) respectively.

**Results:**

Early intervention did not significantly reduce all-cause mortality or the incidence of MI compared with delayed intervention. The frequency of revascularization and major bleeding were also similar between the two groups. A significant reduction was observed for the incidence of recurrent ischemia in early intervention group. Further analyses confirmed those findings across both short-term follow-up (30 days) and mid-to-long-term follow-up (180 days to 5 years). TSA provided additional evidence supporting the protective benefit of early intervention for recurrent ischemia but not for others.

**Conclusions:**

For patients with NSTE-ACS, early invasive treatment does not reduce all-cause mortality or incidence of MI but is associated with a lower frequency of recurrent ischemia.

## Introduction

Non-ST-elevation acute coronary syndrome (*N*STE-ACS) represents a critical spectrum of ischemic heart disease where timely and optimal management is paramount. Current clinical guidelines recommended an early invasive strategy for patients identified as being at high risk, an approach aimed to preventing subsequent ischemic events through prompt revascularization (1, 2). The rational for this strategy is supported by emerging evidence which demonstrates that early intervention, particularly when combined with potent triple antiplatelet therapy, can significantly reduce the incidence of spontaneous cardiac events. This benefit is considered substantial enough to outweigh the heightened risk of periprocedural complications associated with earlier percutaneous coronary intervention (PCI) (3–5).

Conversely, delaying PCI for an extended period has been consistently linked to a higher incidence of spontaneous adverse cardiac events. Furthermore, a growing body of research, including recent studies, has demonstrated that performing coronary angiography shortly after hospital admission can improve clinical outcomes (6). This evidence strengthens the case for a proactive, early invasive approach.

However, the optimal time of intervention remains a subject of ongoing debate, Despite the established benefits of a routine invasive approach, the risks of PCI performed during the dynamic and unstable early phase of acute coronary syndrome (ACS) are not fully elucidated (7). In contrast to the evidence for early action, other studies propose a potential advantage to delayed intervention. This alternative perspective suggests that in patients stabilized with intensive antithrombotic therapy, postponing PCI may minimize procedure-related risks and allow for the revascularization of more stable plaques, potentially leading to more durable results (8, 9).

This persisting clinical equipoise underscores the necessity for a comprehensive synthesis of the available evidence. Therefore, in the current study, we systematically evaluate the therapeutic impact of the timing of invasive interventions in high-risk NSTE-ACS patients through a meta-analysis. Our findings aim to inform clinical decision-making by clearly delineating the risk-benefit profile of early vs. delayed invasive strategies.

## Methods

### Search strategy

We systematically searched the PubMed and EMBASE databases for randomized controlled trials (RCTs) published up to October 14 of 2024, that compared clinical outcomes between early and delayed invasive strategies in patients with NSTE-ACS. The search strategy incorporated Medical Subject Heading (MeSH) and keywords, including “non-ST-elevation acute coronary syndrome”, “percutaneous coronary intervention”, “balloon angioplasty”, “randomized controlled trial”, “randomised” and “randomized”. Additional free-text terms, such as “early invasive strategy” and “delayed invasive strategy”, were also included to ensure comprehensive retrieval.

### Study selection criteria

Studies were included according to the following criteria: (1) enrolled patients diagnosed with NSTE-ACS; (2) directly compared early vs. delayed invasive strategies; (3) defined early intervention as catheterization performed within 24 h; and (4) defined delayed intervention as catheterization performed at or beyond 24 h.

### Study screening and data extraction

Two investigators (Y.W and G.X.Z) independently performed study selection, risk-of-bias assessment, and data extraction. Any disagreements were resolved through consensus. The primary endpoints were all-cause mortality or myocardial infarction (MI), while secondary endpoints included recurrent ischemia, revascularization or major bleeding. The risk of bias for each trial was evaluated using the Cochrane Collaboration's recommended tool (10), and publication bias was assessed visually with funnel plots (10).

### Definition of early vs. delayed intervention

To synthesize evidence across trials with varing protocols, we pre-defined the intervention timing based on the median time from admission or randomization to cardiac catheterization. Consistent with major international guidelines (1, 35), the early invasive strategy was defined as a median time to catheterization of less than 24 h, while the delayed invasive strategy was defined as a median time of 24 h or greater. While the specific protocols within these categories varied (e. g., “immediate” vs. “within 12 h” in the early group), this 24 h dichotomization provides a clinically pertinent and consistent benchmark for our analysis.

### Statistical analyses

#### Sensitivity and heterogeneity analysis

Sensitivity analyses were performed by systematically excluding individual studies to evaluate their impact on the pooled estimates. All outcomes were analyzed according to the intention-to-treat principle. Relative risks (RRs) were calculated using both the DerSimonian and Laird random-effects model and the Mantel-Haenszel fixed-effects model (11, 12). Heterogeneity was quantified with the I² statistic, where I² < 25% represented low heterogeneity and I² > 75% indicated substantial heterogeneity (13). The fixed-effects model was employed when the *p*-value for heterogeneity exceeded 0.05; otherwise, the random-effects model was applied. All analyses were conducted using STATA version 11 (StataCorp), with statistical significance defined as a two-tailed *p*-value < 0.05, and results presented with 95% confidence intervals (CIs).

#### Trial sequential analysis (TSA)

To control the risks of type I and II errors in our cumulative meta-analysis, we performed trial sequential analysis using TSA software (version 0.9 Beta) (14–16). The analysis was configured with the following parameters: (1) a 25% reduction in relative risk, representing a clinically meaningful effect size in cardiovascular research; (2) a two-sided alpha of 5% and statistical power of 80%; and (3) an information size adjusted for diversity. Monitoring boundaries were constructed according to the Lan-DeMets method. If the cumulative Z-curve failed to cross either the efficacy or futility boundary, the evidence was considered insufficient and additional trials would be needed; conversely, boundary crossing provided firm evidence to support the conclusion.

#### PRISMA 2020 statement

This study was conducted and reported following the Preferred Reporting Items for Systematic Reviews and Meta-Analyses (PRISMA) 2020 statement (17). The completed checklist is available in Supplementary File S1.

## Results

### Study selection and characteristics

**Figure 1:**
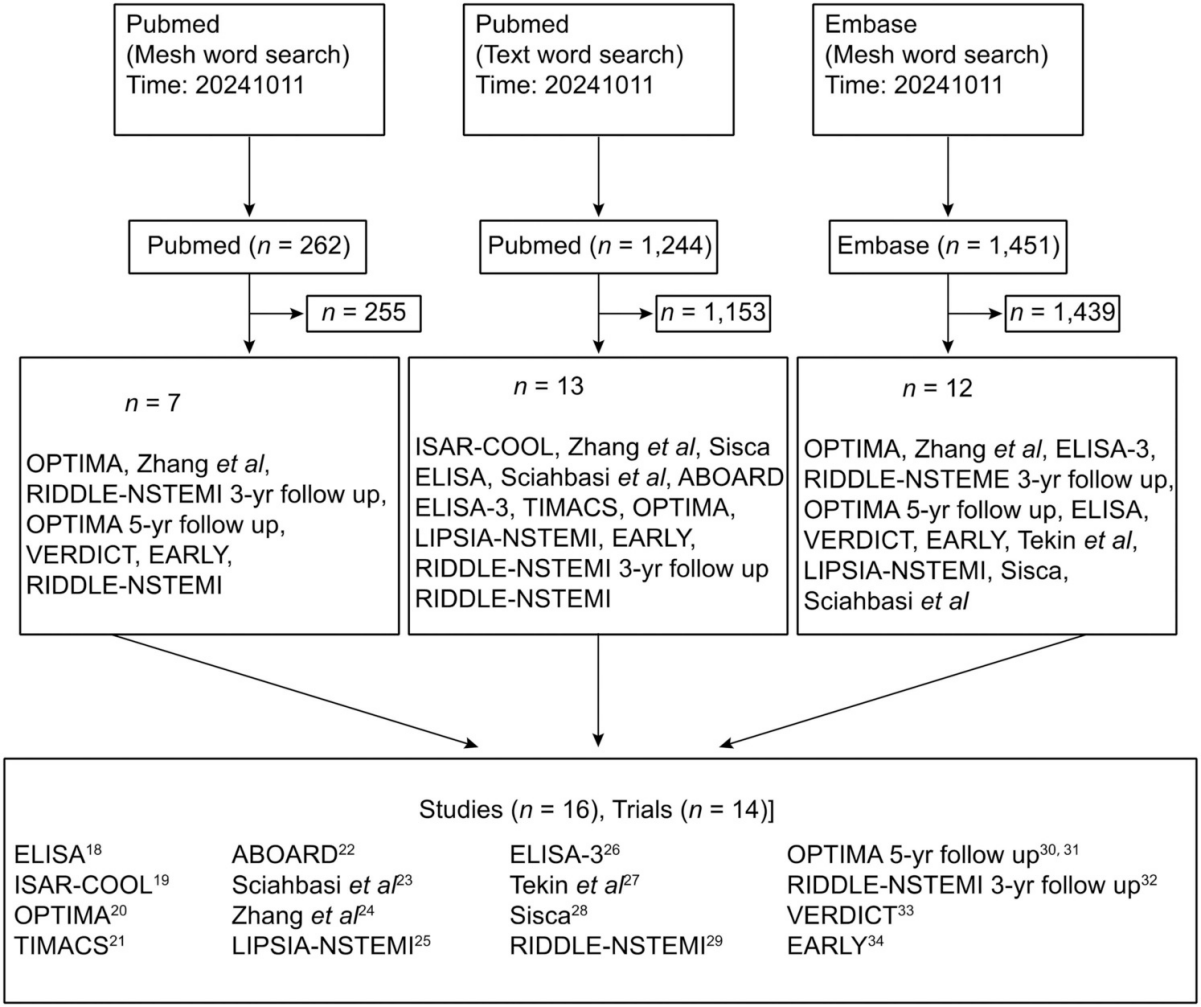
Study selection flow diagram.

The initial screen identified 15 trials that met the selection criteria. One study was subsequently excluded due to methodological limitations, resulting in 14 RCTs being included in the final meta-analysis (18–34) (Figure 1). Detailed characteristics of the included studies are summarized in Table 1.

**Table 1:** Summary of study design and patient characteristics.

No.	Author/Study/Year	Median Time of Catheterization, h		Patients, *n*		Age, years		NSTEMI/Unstable angina		GRACE risk score		TIMI risk score		Primary endpoint	Secondary endpoint	Follow-up
		EDS	DS	EDS	DS	EDS	DS	EDS	DS	EDS	DS	EDS	DS			
1	ELISA (18)	6	50	109	111	63 ± 11	65 ± 11	NA		NA		NA		All-cause mortality and recurrent myocardial infarction were evaluated at the 30-day follow-up point	Major bleeding	30 days
2	ISAR-COOL (19)	2.4	86	203	207	70 (60–77)	70 (62–77)	NA		134 ± 66.0	140 ± 67.6	NA		The 30-day follow-up measured the aggregate incidence of major myocardial infarction and all-cause mortality	Major bleeding	30 days
3	OPTIMA (20)	0.5	25	73	69	63 ± 12	62 ± 12	NA		NA		NA		The primary endpoint was a 30-day composite outcome including death, non-fatal myocardial infarction, and unplanned revascularization following enrollment	Secondary endpoint included each component of the primary composite outcome, as well as revascularization considered separately.	30 days, 6 months
														Major bleeding		
4	TIMACS (21)	14	50	1,593	1,438	65.0	65.7	NA		NA		NA		The primary outcome was defined as the initial incidence of a composite event comprising death, new myocardial infarction, or stroke within a 6-month period	Secondary outcomes included death, myocardial infarction, or refractory ischemia at 6 months, as well as the occurrence of major bleeding	6 months
5	ABOARD (22)	1.1	20.5	175	177	65 ± 12	65 ± 12	NA		NA		NA		The primary endpoint was defined as the highest troponin level recorded during the hospital stay	The main secondary endpoint was a composite of death, myocardial infarction, or urgent revascularization at 1-month follow-up, along with the incidence of major bleeding	1 month
6	Sciahbasi *et al.* (23)	5	24	27	27	58.8 ± 9.4	59.7 ± 9.8	NA		NA		NA		The study aimed to assess the impact of the two reperfusion strategies on microvascular injury and myocardial perfusion-evaluated myocardial contrast echocardiography (MCE) and myocardial blush grade (MBG), respectively-as well as on infarct size, determined by myocardial enzyme levels	Outcomes included mortality, stent thrombosis, and revascularization of the target vessel. Key echocardiographic metrics, including left ventricular ejection fraction (LVEF)	1 year
7	Zhang *et al. (*24)	9.3	49.9	446	369	61.22 ± 7.88	61.65 ± 6.61	9/31	9/31	NA		NA		The primary endpoint consisted of a composite outcome including death, myocardial infarction, and stroke	The secondary endpoint comprised a composite of death, myocardial infarction, and refractory ischemia, along with the occurrence of major bleeding	180 days
8	LIPSIA-NSTEMI (25)	1.1	18.3	200	200	68 (58–76)	70 (59–79	NA		133 (115–154)	137 (112–160)	NA		The primary outcome was defined as the highest level of creating kinase-myocardial band (CK-MB) activity recorded during the initial hospitalization	Key second clinical outcomes included several composite measures assessed over a 6-month period: (i) death and non-fatal myocardial infarction; (ii) death, non-fatal infarction ischemia; and (iii) death, non-fatal infarction, refractory ischemia, and re-hospitalization due to unstable angina	6 months
															Major bleeding	
9	ELISA-3 (26)	2.6	54.9	269	265	72.1 (65.5–78.4)	71.8 (62.5–78.4)	NA		136 (118–154)	133 (117–154)	NA		The primary endpoint was the 30-day cumulative incidence of death, reinfarction, and/or recurrent ischemia	Secondary endpoint included enzymatic infarct size, measured by a single cardiac troponin T level obtained at 72–96 h post-admission or at discharge, and the proportion of patients who showed no CK-MB elevation during hospitalization	30 days
															Major bleeding	
10	Tekin *et al.* (27)	<24	24–72	69	62	58.1 ± 10.3	55.6 ± 10.1	NA		NA		4.10 ± 0.85	4.19 ± 1.02	Major adverse cardiac events were defined to include LVEF reduction, recurrent myocardial infarction, hospital readmission for cardiac causes, and all-cause mortality		3 months
11	SISCA (28)	2.8	20.9	83	86	63.9 (55.6–72.5)	66.5 (57.5–75.8)	NA		NA		3 (2–4)	4 (3–4)	The primary endpoint was the 30-day cumulative occurrence of death, myocardial infarction, or the need for urgent revascularization	Secondary endpoint included: unsuccessful (1) outcomes associated with delayed intervention; (2) revascularization procedures, including CABG, performed between 6 h and 30 days; (3) peak troponin I levels; (4) left ventricular ejection fraction (LVEF) at discharge; (5) duration of hospitalization; (6) incidence of major or minor bleeding; and (7) long-term all-cause mortality	4.1 years (3.3–5.4)
12	RIDDLE-NSTEMI (29)	1.4 (1.00–2.24)	61.0 (35.8–85.0)	162	161	60.5 (52–69)	63.0 (55–71)	NA		131 (115–144)	129 (115–150)	3.5 (3–4)	4 (3–4.5)	The primary outcome was a 30-day composite of all-cause mortality or newly occurring myocardial infarction	The secondary endpoint comprised a composite of death, myocardial infarction, and refractory ischemia, along with the occurrence of major bleeding	180 days
13	The OPTIMA trial 5-yr follow-up (30, 31)	0.5	25	73	69	63 ± 12	62 ± 12	NA		NA		NA		The primary endpoint consisted of a composite of death and spontaneous myocardial infarction, with the latter defined as an MI occurring more than 30 days post-randomization	Secondary outcomes encompassed separate assessments of mortality, spontaneous MI, and re-PCI	5 years
14	The RIDDLE-NSTEMI study 3-yr follow-up (32)	1.4	61	162	161	60.5 (52–69)	63.0 (55–71)	NA		NA		NA		The primary endpoint was the composite of death or new myocardial infarction (MI)	Death, MI, Major bleeding	3 years
15	VERDICT (33)	4.7 (3.0–12.2)	61.6 (39.4–87.8)	1075	1072	63.6 ± 12.1	63.6 ± 12.5	NA		141.3 ± 29.8	140.8 ± 31.4	NA		The primary endpoint comprised a composite of all-cause mortality, non-fatal recurrent myocardial infarction, hospitalization due to refractory ischemia or admission for heart failure	Endpoint included complications related to invasive procedures during the index hospitalization, such as procedure-related death, bleeding, non-fatal acute myocardial infarction, stoke, and transient ischemic attack, as well as the occurrence of any of the following events post-randomization: death, non-fatal acute MI, hospitalization for refractory ischemia, repeat coronary revascularization, or admission for heart failure	4.3 years (interquartile range 4.1–4.4 years)
16	EARLY (34)	Under 2 (0–1)	18 (12–23)	346	362	65.0 ± 12.4	65.5 ± 13.0	238/23	244/26	123.4 ± 35.1	121.2 ± 31.9	NA		The primary outcome was defined as a composite of cardiovascular death and/or a recurrent ischemic event necessitating urgent revascularization within one month after randomization	Secondary endpoint was as follows: (1) the incidence of cardiovascular death and/or recurrent ischemic events requiring urgent revascularization; (2) the rat of cardiovascular death; (3) the incidence of all-cause mortality; (4) the frequency of recurrent ischemic episodes requiring urgent revascularization; (5) the occurrence of non-fatal myocardial infarction; (6) the rat of bleeding events classified by BARC criteria; (7) the peak troponin level recorded during the index hospitalization; (8) the length of hospital stay	1 month

CABG, coronary artery bypass grafting; DS, delayed strategy; EDS, early delayed strategy; MACE, major adverse cardiovascular events; MI, myocardial infarction; PCI, percutaneous coronary intervention; NSTEMI, non-ST-segment elevation myocardial infarction; NSTE-ACS, non-ST-segment elevation acute coronary syndrome.

The final analysis comprised a total of 9,436 patients, with 4,830 (51.2%) assigned to the early intervention group and 4,606 (48.8%) to the delayed intervention group. The follow-up duration across the studies ranged from 1–60 months. All included trials provided data for the systematic evaluation of safety and efficacy outcomes.

### Quality assessment

Quality assessment results are summarized in Supplementary Table S1. Funnel plots for primary outcomes (all-cause mortality and MI) demonstrated no significant asymmetry, suggesting an absence of publication bias (Supplementary Figure S1). Sensitivity analyses confirmed consistent effect sizes for primary endpoints (Supplementary Figure S2 and Supplementary Table S2), underscoring the robustness of our findings.

### Primary and secondary outcomes

#### All-cause mortality and MI

**Figure 2:**
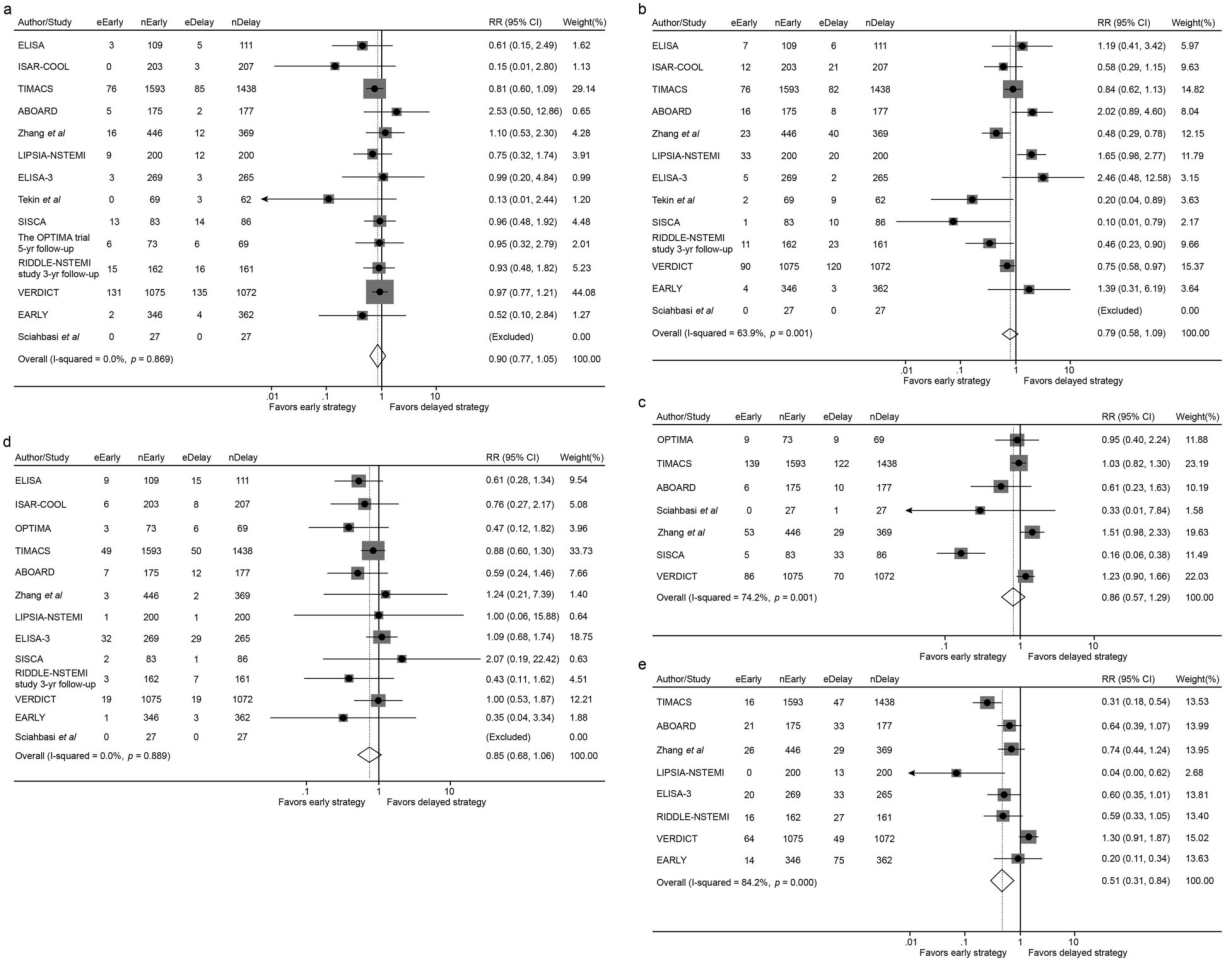
Forest plot comparing all-cause mortality **(a)**, the incidence of myocardial infarction **(b)**, overall revascularization **(c)**, major bleeding **(d)** and recurrent ischemia **(e)** between early and delayed invasive strategies for all enrolled trials/studies during the whole follow-up period. RR represents for relative risk.

- All-Cause Mortality: No statistically significant difference was observed between early and delayed intervention strategies (5.78% vs. 6.51%; RR 0.90, 95% CI 0.77–1.05, *p* = 0.162) (Figure 2a).
- MI: Similarly, the incidence of MI showed no significant differences between groups (5.89% vs. 7.06%; RR 0.79, 95% CI 0.58–1.09, *p* = 0.159) (Figure 2b).

#### Revascularization and major bleeding

- Revascularization: Revascularization rates were comparable between the early and delayed intervention groups (8.58% vs. 8.46%; RR 0.86, 95% CI 0.57–1.29, *p* = 0.458) (Figure 2c).
- Major Bleeding: No significant difference was identified in major bleeding events between the two groups (2.84% vs. 3.37%; RR 0.85, 95% CI 0.68–1.06, *p* = 0.151) (Figure 2d).

#### Recurrent ischemia

- Early intervention is significantly associated with lower incidence of recurrent ischemia compared with delayed intervention (4.15% vs. 7.57%; RR 0.51, 95% CI 0.31–0.84, p = 0.009) (Figure 2e).

### Subgroup analysis by follow-up duration

#### Outcomes in the short-term (30-day) follow-up subgroup

**Figure 3:**
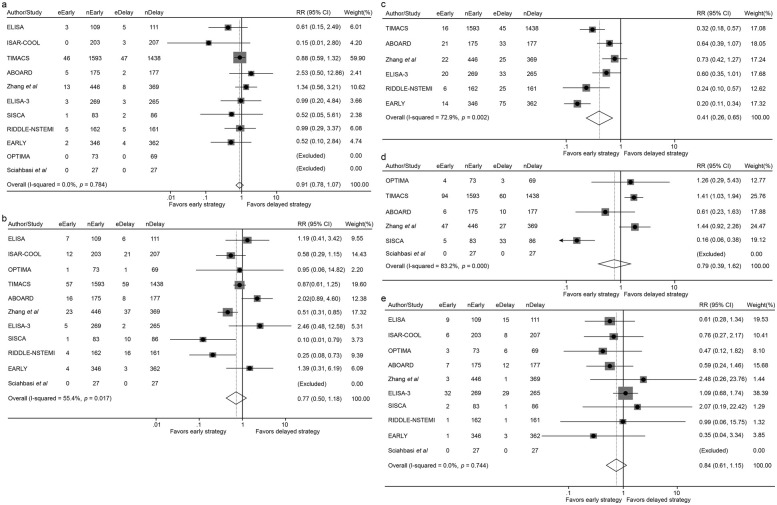
Forest plot comparing all-cause mortality **(a)**, the incidence of myocardial infarction **(b)**, recurrent ischemia **(c)**, overall revascularization **(d)** and major bleeding **(e)** between early and delayed invasive intervention strategies in the short-term follow-up subgroup.

**Figure 4:**
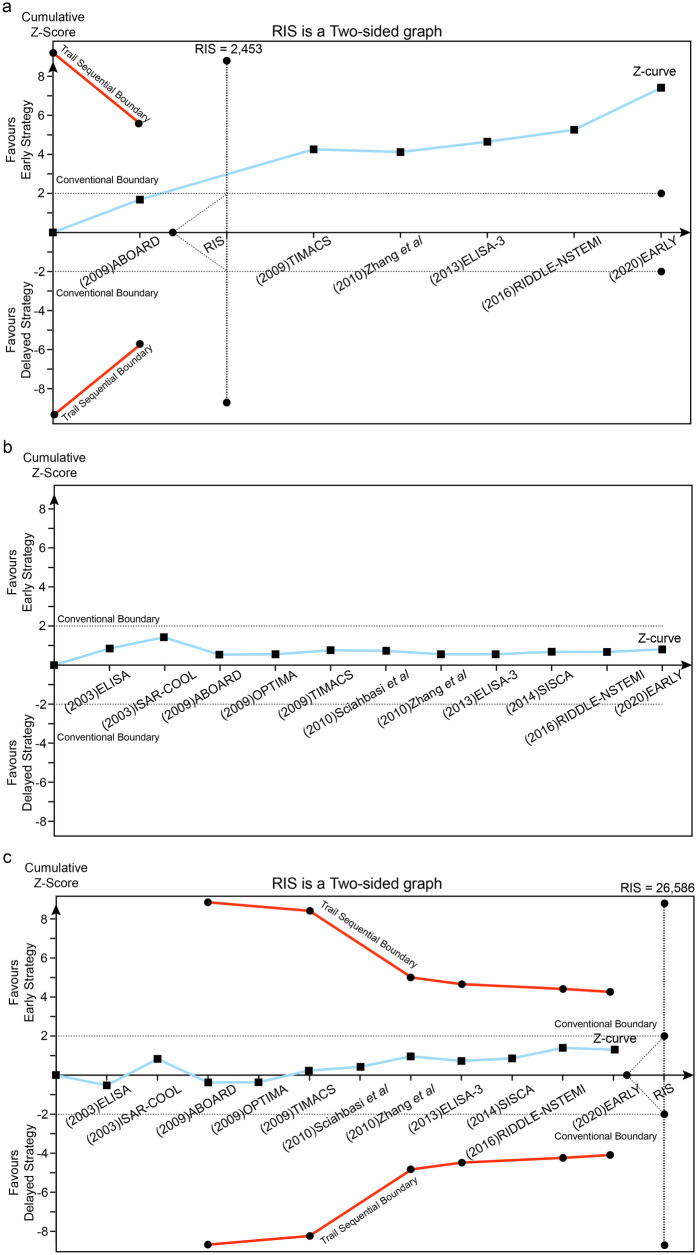
Trial sequential analysis (TSA) assessing recurrent ischemia **(a)**, all-cause death **(b)** and MI **(c)** between early and delayed invasive strategies across the short-term follow-up subgroup.

In the short-term follow-up analysis, no significant differences were observed between early and delayed intervention for all-cause mortality (2.24% vs. 2.44%; RR 0.91, 95% CI 0.67–1.24, *p* = 0.546), MI (3.73% vs. 4.98%; RR 0.77, 95% CI 0.50–1.18, *p* = 0.228), revascularization (6.51% vs. 6.14%; RR 0.79, 95% CI 0.39–1.62, *p* = 0.521) or major bleeding (3.38% vs. 4.14%; RR 0.84, 95% CI 0.61–1.15, *p* = 0.271). However, patients in the early intervention group demonstrated a significant reduction in recurrent ischemia compared with that of the delayed intervention group (3.31% vs. 8.54%; RR 0.41, 95% CI 0.26–0.65, *p* < 0.001) (Figures 3, 4 and Table 2).

**Table 2:** Outcomes in the short-term (30-day) follow-up subgroup.

Outcome	Early intervention	Delayed intervention	RR (95% CI)	*p*-Value
Mortality	2.24%	2.44%	0.91 (0.67–1.24)	0.546
MI	3.73%	4.98%	0.77 (0.50–1.18)	0.228
**Recurrent Ischemia**	**3** **.** **31%**	**8** **.** **54%**	**0.41** **(0.26–0.65)**	**<0** **.** **001**
Revascularization	6.51%	6.14%	0.79 (0.39–1.62)	0.521
Major Bleeding	3.38%	4.14%	0.84 (0.61–1.15)	0.271

Bold indicates a significant reduction in recurrent ischemia with early intervention.

### Outcomes in the mid-to-long-term (≥180 days to 5 years) follow-up subgroup

**Figure 5:**
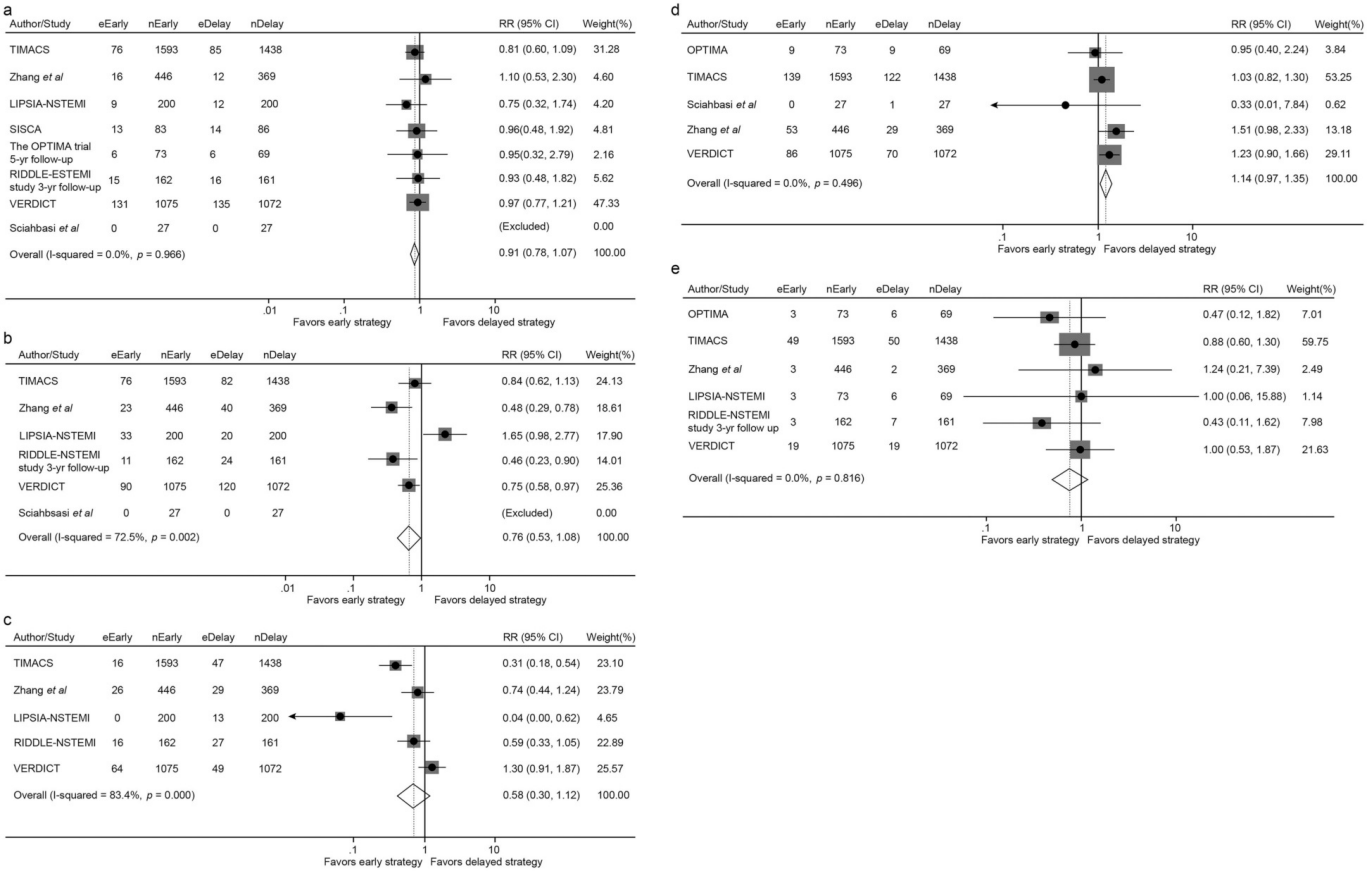
Forest plot comparing all-cause mortality **(a)**, the incidence of myocardial infarction **(b)**, recurrent ischemia **(c)**, overall revascularization **(d)** and major bleeding **(e)** between early and delayed invasive intervention strategies in the mid-to-long-term follow-up subgroup.

**Figure 6:**
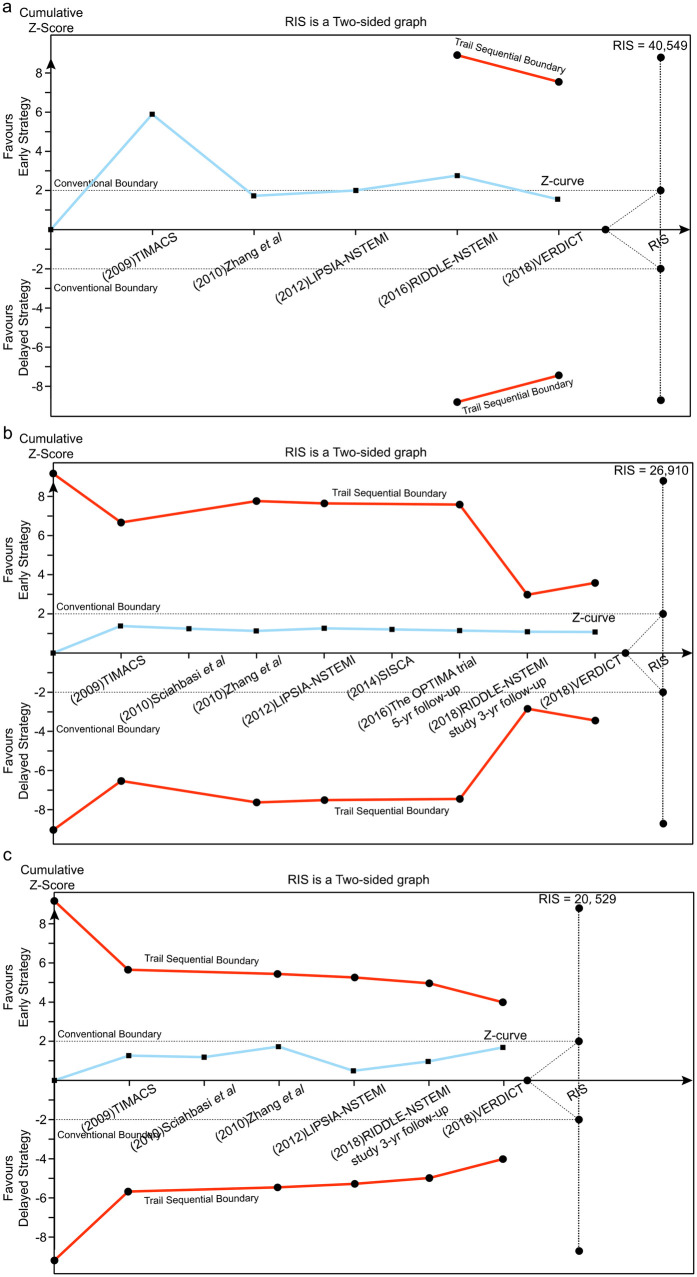
Trial sequential analysis (TSA) assessing recurrent ischemia **(a)**, all-cause death **(b)** and MI **(c)** between early and delayed invasive strategies across the mid-to-long-term follow-up subgroup.

In the mid-to-long-term follow-up subgroup, no significant differences were observed between early and delayed intervention for all-cause mortality (7.27% vs. 8.18%; RR 0.91, 95% CI 0.78–1.07, *p* = 0.257), MI (6.65% vs. 8.75%; RR 0.76, 95% CI 0.53–1.08, *p* = 0.124), revascularization (8.93% vs. 7.77%; RR 1.14, 95% CI 0.97–1.35, *p* = 0.117), or major bleeding (2.19% vs. 2.57%; RR 0.85, 95% CI 0.63–1.16, *p* = 0.306). A non-significant trend toward reduced recurrent ischemia was observed with early intervention (3.51% vs. 5.09%; RR 0.58, 95% CI 0.30–1.12, *p* = 0.105) (Figures 5, 6 and Table 3).

**Table 3:** Outcomes in the mid-to-long-term (≥180 days to 5 years) follow-up subgroup.

Outcome	Early intervention	Delayed intervention	RR (95% CI)	*p*-Value
Mortality	7.27%	8.18%	0.91 (0.78–1.07)	0.257
MI	6.65%	8.75%	0.76 (0.53–1.08)	0.124
**Recurrent Ischemia**	**3** **.** **51%**	**5** **.** **09%**	**0.58** **(0.30–1.12)**	**0** **.** **105**
Revascularization	8.93%	7.77%	1.14 (0.97–1.35)	0.117
Major Bleeding	2.19%	2.57%	0.85 (0.63–1.16)	0.306

Bold indicates a non-significant trend toward reduced recurrent ischemia was observed with early intervention.

#### Trial sequential analysis (TSA)

Using a prespecified 25% RR reduction threshold, TSA demonstrated sufficient evidence that early intervention reduces recurrent ischemia, as the cumulative Z-curve crossed the predefined monitoring boundary. In contrast, there was insufficient evidence to establish an effect of early intervention on all-cause mortality or MI across the analyzed subgroups.

## Discussion

This comprehensive meta-analysis and trial sequential analysis (TSA) of 14 RCTs provides compelling evidence regarding the timing of invasive strategies in NSTE-ACS management. Our principal finding is that an early invasive approach, compared to a delayed strategy, does not confer a significant benefit in reducing the hard endpoints of all-cause mortality or MI at the study level. However, it consistently demonstrateds a powerful and robust advantage in significantly reducing the incidence of recurrent ischemia, a finding cofirmed by TSA. This central result underscores a critical distinction in the management of NSTE-ACS: while an early invasive strategy effectively mitigates ongoing ischemia, its translation into a survival or hard event benefit remains elusive within the current evidence base.

The interpretation of our findings requires integration into the context of the existing, and seemingly conflicting, body of literature. Our results align with several key trials included in our analysis, such as OPTIMA (20) and ABOARD (22), which found no significant advantage for early intervention in reducing mortality or MI. The OPTIMA (20) trial, for instance, reported a higher incidence of MI with immediate PCI compared to a strategy deferred for 24–48 h. Conversely, other landmark trials like TIMACS (21) and RIDDLE-NSTEMI (29) demonstrated benefits for an early approach, particularly in reducing composite endpoints that included refractory ischemia. These apparent discrepancies are not necessarily contradictory but rather highlight the heterogeneity in trial designs, patient populations, and definitions of “early” vs. “delayed” intervention, which ranged from immediate to over 72 h.

A pivotal factor reconciling these divergent results is risk stratification. Our findings are consistent with the well-established paradigm that the benefits of an invasive strategy are not uniform across the NSTE-ACS spectrum. As supported by the TIMACS (21) trial and others, high-risk patients-such as those with refractory angina, hemodynamic instability, or significant dynamic electrocardiography (ECG) changes-derive the greatest benefit from an expedited invasive approach. This is further corroborated by guidelines from the European Society of Cardiology and the American Heart Association/American College of Cardiology, which recommend a very early invasive strategy (within 2–24 h) for this high-risk subgroup. The observed reduction in recurrent ischemia in our analysis likely reflects the successful application of this principle in a substantial portion of the enrolled patients across the trials.

Recently, the incorporation of high-sensitivity cardiac troponin (hs-cTn) assays into clinical practice has fundamentally refined risk stratification and the subsequent timing of ivasive strategies in patients with NSTE-ACS. Those assays enable rapid and accurate patient triage through validated 0/1 h or 0/2 h algorithms, effectively distinguishing those at very low risk who can be safely discharged form those with confirmed myocardial injury who require inpatient management (35, 36). This dynamic, hs-cTn-based risk assessment moves beyond static, one-time evaluations and allows for the categorization of patients into a continuous risk spectrum. Comsequently, the decision for an early (within 24 h), delayed (within 24–72 h), or selective invasive strategy can be precisely individualized, moving away from a on-size-fits-all approach to one guided by the acuity and magnitude of myocardial injury (35). It is crucial to emphasize that the optimal timing of revascularization, while critical, is only one component of comprehensive ACS care. Regardless of the chosen interventional timing, achieving the best possible prognosis hinges on the concurrent initiation and maintenance of guideline-directed medical therapy (GDMT). This includes potent dual antiplatelet therapy-with a preference for ticagrelor or prasugrel over clopidogrel where appropriate-and intensive lipid-lowering strategies utilizing high-intensity statins, often in combination with non-statin agents like ezetimibe or PCSK9 inhibitors to achieve stringent LDL-C targets. Furthermore, a holistic management approach must be reinforced by structured cardiac rehabilitation programs, which are integral to improving functional status and long-term cardiovascular outcomes. Therefor, the modern management of NSTE-ACS, informed by hs-cTn, integrates precise risk-stratified timing of intervention with robust, multifaceted pharmacological and non-pharmacological secondary prevention stratigies to optimize patient prognosis (37, 38).

Our subgroup analyses provide additional insights into the timing dilemma. The short-term (30-day) outcomes revealed no mortality benefit but demonstrated significant reduction in recurrent ischemia with early intervention. In the mid-to-long-term follow-up (≥180 days to 5 years), early intervention showed non-significant trends toward reduced recurrent ischemia without increased risks of major bleeding or other complications. Those findings suggest that the primary benefit of early intervention may lie in rapid ischemia resolution rather than hard endpoint modification.

In conclusion, based on our meta-analysis and supported by the broader scientific consensus, a one-size-fits-all approach to the timing of intervention in NSTE-ACS is obsolete. The decision should be individualized, pivoting on three key axes: the patient's risk profile (informed by contemporary tools like hs-cTn and GRACE score), the ongoing ischemic burden, and the individual bleeding risk. An early invasive intervention is strongly indicated for high-risk patients, primarily to abate recurrent ischemia, while a more delayed approach remains a safe and reasonable option for stabilized, lower-to-intermediate-risk individuals. Future research should focus on large-scale randomized trials employing standardized, contemporary timing protocols aligned with modern biomarker-guided stratification and GDMT to definitively address the impact on mortality and MI outcomes.

### Study limitations

A critical aspect of interpreting our results is the substantial heterogeneity observed among the included studies, which precludes simplistic conclusions. This heterogeneity is not a limitation of the meta-analysis but rather a reflection of the true clinical and methodological diversity across the trials. There indeed several key sources of this variability: (1) patient risk stratification: The enrolled populations varied in their baseline risk. Earlier trials often included a broader mix of patients, while more contemporary ones increasingly focused on high-risk cohorts defined by biomarkers like elevated troponins. The conflicting results between trials like TIMACS (which showed benefit in high-risk subgroups) and OPTIMA (which did not) can be largely attributed to these differences in the underlying risk profiles of their study populations. (2) Definitions of early vs. delayed intervention: The protocols for timing were not standardized. The definition of “early” ranged from immediate (within 2 h) to within 24 h, while “delayed” could mean the next day (24–48 h) or up to 72 h or even longer in some cases.

## Conclusions

Based on the current meta-analysis of 14 randomized controlled trials, we conclude that in patients with NSTE-ACS, an early invasive strategy does not confer a significant reduction in all-cause mortality or the incidence of myocardial infarction compared with a delayed intervention approach. Nevertheless, early invasive management is consistently associated with a marked decrease in recurrent ischemia, a benefit that was further corroborated by trial sequential analysis. Those findings were consistent across short-term and mid-to-long-term follow-up periods, with no increased risk of major bleeding or other procedure-related complications observed in the early intervention group.

Given those results, we suggest that the decision to pursue an early vs. delayed invasive strategy should be individualized, taking into account the patient's clinical stability, ischamic burden, and overall risk profile. Early intervention maybe prioritized in those with high-risk features or ongoing ischemia, whereas a more delayed approach remains a reasonable option in stabilized individuals. Further large-scale studies are warranted to explore the effects of timing on other clinical endpoints and in specific patient subgroups.

## Data Availability

The original contributions presented in the study are included in the article/Supplementary Material, further inquiries can be directed to the corresponding authors.
