# Supplementary figures and images for: Assessing the timing of invasive intervention in NSTE-ACS: insights from a meta-analysis and sequential trial evaluation
Source: Front Cardiovasc Med. 2025 Nov 20;12:1712137. doi: 10.3389/fcvm.2025.1712137 (PMC12675449; doi:10.3389/fcvm.2025.1712137)

**Supplementary Fig1.** Funnel plot showing the pseudo 95% confidence limits.

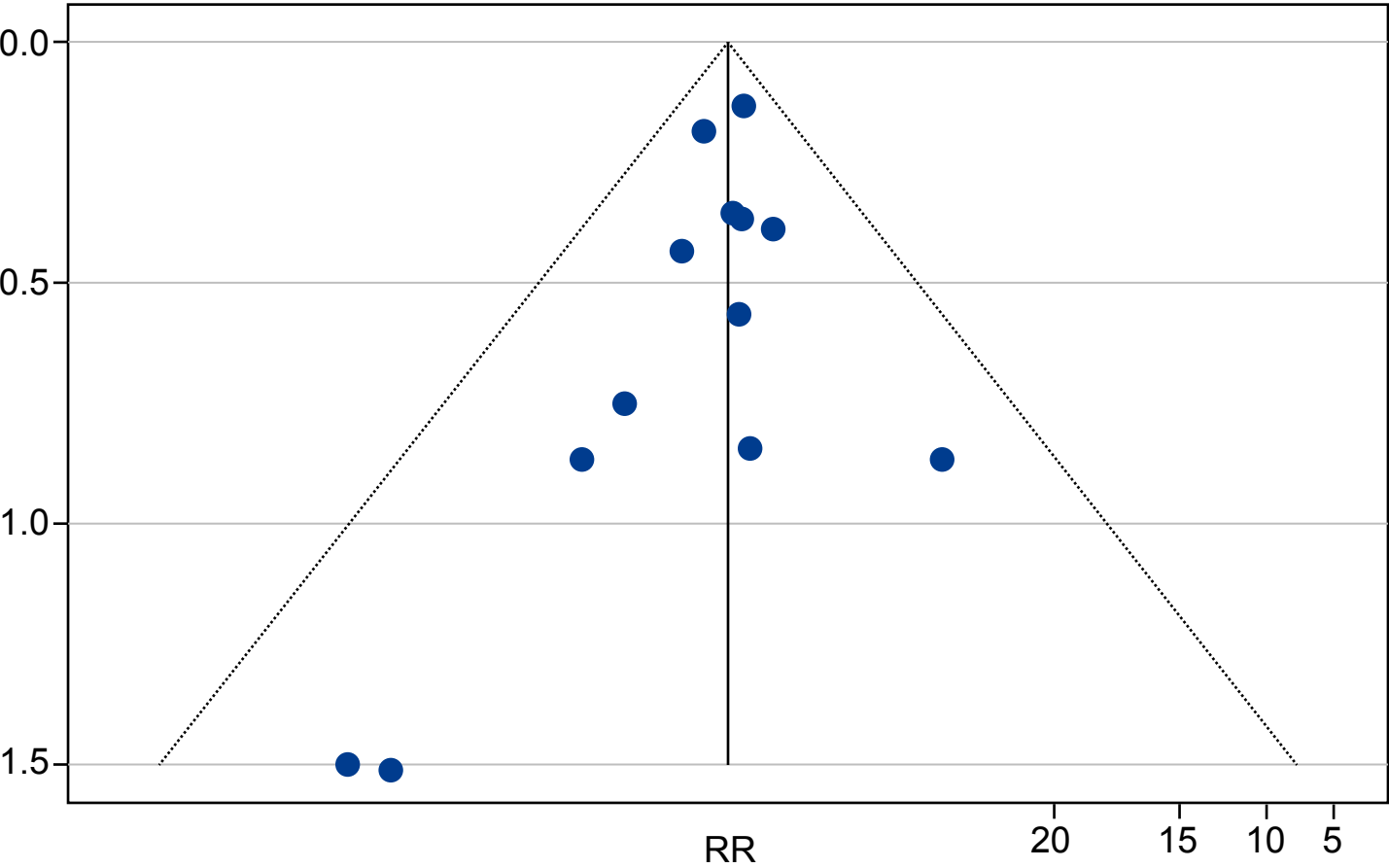

Supplement: Supplementary file 1 [file Datasheet1.pdf]
