# Supplementary material for: Assessing the timing of invasive intervention in NSTE-ACS: insights from a meta-analysis and sequential trial evaluation
Source: Front Cardiovasc Med. 2025 Nov 20;12:1712137. doi: 10.3389/fcvm.2025.1712137 (PMC12675449; doi:10.3389/fcvm.2025.1712137)
Supplement: Supplementary file 3 [file Table1.doc]

**Supplementary Table 1.** Bias analysis of all enrolled trials/studies.

| Study/author/reference | Random sequence generation  (Selection bias) | Allocation concealment  (Selection bias) | Blinding of participants and personnel  (Performance bias) | Blinding of outcome assessment  (Detection bias) | Incomplete outcome data  (Attrition bias) | Selective reporting  (Reporting bias) |
| --- | --- | --- | --- | --- | --- | --- |
| ELISA^18^ |  |  |  |  |  |  |
| ISAR-COOL^19^ |  |  |  |  |  |  |
| OPTIMA^20^ |  | 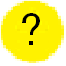 |  |  |  |  |
| TIMACS^21^ |  |  |  |  |  |  |
| ABOARD^22^ |  |  |  |  |  |  |
| Sciahbasi, *et al*^23^ |  |  |  |  |  |  |
| Zhang, *et al*^24^ |  | 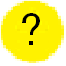 | 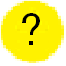 |  |  |  |
| LIPSIA-NSTEMI^25^ |  | 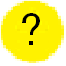 |  |  |  |  |
| ELISA-3^26^ |  |  |  |  |  |  |
| Tekin *et al*^27^ |  |  |  |  |  |  |
| Sisca^28^ |  | 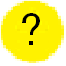 |  |  |  | 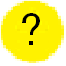 |
| RIDDLE-NSTEMI^29^ |  |  |  |  |  |  |
| OPTIMA-5-yr follow up^30,31^ |  |  |  |  |  |  |
| RIDDLE-NSTEMI-3-yr follow up^32^ |  |  |  |  |  |  |
| VERDICT^33^ |  |  |  |  |  |  |
| EARLY^34^ |  |  |  |  |  |  |
| low risk of bias; 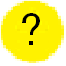 unclear risk of bias; 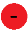 high risk of bias | | | | | | |
