# Supplementary material for: Assessing the timing of invasive intervention in NSTE-ACS: insights from a meta-analysis and sequential trial evaluation
Source: Front Cardiovasc Med. 2025 Nov 20;12:1712137. doi: 10.3389/fcvm.2025.1712137 (PMC12675449; doi:10.3389/fcvm.2025.1712137)
Supplement: Supplementary file 4 [file Table2.docx]

**Supplementary Table 2.** Assessment of the influence of each study on the overall effect for all-cause death

| Trials/Studies | Estimate | 95% confidence interval |
| --- | --- | --- |
| ELISA^18^ | 0.90 | 0.77 1.05 |
| ISAR-COOL^19^ | 0.90 | 0.77 1.05 |
| TIMACS^21^ | 0.93 | 0.78 1.12 |
| ABOARD^22^ | 0.88 | 0.76 1.03 |
| Sciahbasi *et al*.^23^ | 0.90 | 0.77 1.05 |
| Zhang *et al*.^24^ | 0.89 | 0.76 1.04 |
| LIPSIA-NSTEMI^25^ | 0.90 | 0.77 1.06 |
| ELISA-3^26^ | 0.90 | 0.77 1.05 |
| Tekin *et al*.^27^ | 0.90 | 0.77 1.06 |
| SISCA^28^ | 0.89 | 0.76 1.05 |
| The OPTIMA trial 5-yr follow-up^30,31^ | 0.89 | 0.76 1.05 |
| RIDDLE-NSTEMI study 3-yr follow-up^32^ | 0.89 | 0.76 1.05 |
| VERDICT^33^ | 0.84 | 0.68 1.04 |
| EARLY^34^ | 0.90 | 0.77 1.05 |
| Combined | 0.90 | 0.77 1.05 |
